# SARS-CoV-2 and the reproductive system: known and the unknown..!!

**DOI:** 10.1186/s43043-020-00046-z

**Published:** 2021-01-07

**Authors:** Indu Sharma, Priti Kumari, Anuradha Sharma, Subhas Chandra Saha

**Affiliations:** 1 Department of Zoology, Panjab University, Chandigarh, 160014 India; 2 Department of Obstetrics & Gynecology, Postgraduate Institute of Medical Education and Research (PGIMER), Chandigarh, 160012 India

**Keywords:** ACE2, Breast milk, COVID-19, Maternal-fetal transmission, Ovary, Reproductive health, SARS-CoV-2, Testes

## Abstract

**Background:**

COVID-19 is the most recent zoonotic outbreak of coronaviruses. Mostly, it invades the cells of the respiratory system by binding to the receptor angiotensin-converting enzyme 2 (ACE2) which is also present in other organs like the kidney, testis, ovaries, breast, heart, and intestine, rendering them prone to be infected. The reproductive potential is a must for the sustenance of any species and it is our prime duty to safeguard the reproductive system of the present generation from such a deadly virus. The previously reported coronaviruses like severe acute respiratory syndrome coronavirus (SARS-CoV) had a detrimental impact on reproductive organs. There is a dearth of sufficient research to provide substantial evidence for the harmful effects of this novel virus on the reproductive system. Hence, our review compiles the knowledge available until now to boost research in this regard and to take the necessary steps in time.

**Main body of abstract:**

Here we tried to compile all the data available on the effect of SARS-CoV-2 on the reproductive system as well as vertical transmission of the virus. All related articles published from February to August 2020 were reviewed and thoroughly analyzed. SARS-CoV-2 has been found to affect the sperm concentration and motility, thus degrading the fertility of males. In females, it is suspected that this virus affects the oocyte quality and ovarian function, resulting in infertility or miscarriage. Traces of SARS-CoV-2 virus have also been found in the breast milk of the infected mothers and the semen of infected males. Vertical transmission of SARS-CoV-2 has also been reported in some cases.

**Conclusion:**

Based on the literature review, SARS-CoV-2 seems to have the potential of affecting both male and female reproductive tracts. This review brings together the findings and observations made in the area of reproductive health during the current pandemic. The reproductive system of the young population is preordained for subsequent disorders, infertility, reduced sperm count, and motility. Therefore, the research and medical practices should focus on possible vulnerability being posed by SARS-CoV-2 to the gametes and future generations. We, hereby, recommend close monitoring of young and pregnant COVID-19 patients concerning reproductive health with utmost priority.

## Background

The disease COVID-19 (coronavirus disease 2019), which occurs due to severe acute respiratory syndrome coronavirus-2 (SARS-CoV-2), was recognized as the global pandemic on March 11, 2020, considering the number of people infected and fatalities caused [1]. Till September 14, 2020, around 29,189,557 confirmed cases of coronavirus and 928,333 deaths have been reported due to SARS-CoV-2 worldwide. In India alone, around 4,846,427 cases were confirmed with 79,754 reported deaths [2]. SARS-CoV-2 shares 76% amino acid homology with the previously reported SARS-CoV and enters the target host cells through a similar route [3]. Viral entry requires the binding of SARS-CoV-2 through its spike (S) glycoprotein to the host receptor angiotensin-converting enzyme 2 (ACE2). The viral entry is further processed by the transmembrane serine protease 2 (TMPRSS2) which cleaves and brings about the conformational change in the spike protein permitting the permanent fusion of the cell membranes of virus and host cell [4, 5]. In broad terms, the SARS-CoV-2 is believed to affect mainly the respiratory system; however, other organs such as kidneys, heart, breasts, intestine, testes, and ovaries, etc., have also been reported as the target organs of SARS-CoV-2 infection [6].

The infection of the reproductive system calls for greater attention because it not only affects the current generation but may also extend up to the future progeny through impaired gametes. Till today, several studies have asserted the effect of SARS-CoV-2 on both male as well as female reproductive system including the vertical transmission of the virus [7, 8] but a definite conclusive report is not yet available, because of the difference in study samples, collection techniques, ethnicity, and above all the pandemic is still active. Besides, we are still ignorant of the fact that what differentiates symptomatic and asymptomatic populations? The objective of the present review is to compile and summarize all the available studies suggesting the potential risk of SARS-CoV-2 on the reproductive system as well as evaluating the effects of the current pandemic with comparison to previous coronavirus outbreaks so that extensive research and timely initiatives can be carried out for the protection of overall reproductive health.

## Main text

Extensive literature exploration was done using PubMed, Google Scholar, Scopus, and MEDLINE databases regarding the role of SARS-CoV-2 on both male and female reproductive systems as well as vertical transmission. The keywords used for our search strategy were SARS-CoV-2, COVID-19, testes, ovaries, breast, placenta, ACE2, and maternal-fetal transmission. Papers published from February 2020 onwards were thoroughly searched in all databases, and for comparison to previously reported SARS coronaviruses, some previous studies were also included. The studies evaluated for this narrative review mainly include case reports, case-control studies, cohort studies, and systematic reviews. The studies published without peer-review were not included in the present review.

### Origin and mode of action of SARS-CoV-2

It is believed that SARS-CoV-2 has been originated from bats, and after undergoing several mutations, it becomes capable of infecting humans [9]. Pangolin is believed to be the intermediate host of the SARS-CoV-2 [10]. A spike protein-containing variable receptor-binding domain (RBD) covers all the SARS-related coronaviruses. Binding of the RBD to angiotensin-converting enzyme 2 (ACE2) receptor facilitates the viral entry into target cells. Genomic sequencing data revealed that the RBD of SARS-CoV-2 is a mutated version of its most closely related virus, RaTG13, sampled from bats (*Rhinolophusaffnis*) [11].

SARS-CoV-2 resembles the previously reported SARS-CoV due to their genetic similarity (76% amino acid homology) as well as their mode of action. In the lungs, binding of SARS-CoV with the angiotensin-converting enzyme 2 (ACE2) receptors triggers a surge of inflammation in the type II pneumocytes [12]. SARS-coronaviruses follow the same mechanism as employed by influenza and human metapneumovirus for entry in the host cell where the spike protein of SARS-corona virus binds to the ACE2 receptor and forms a complex which is proteolytically processed by TMPRSS2 (type 2 transmembrane protease) resulting in cleavage of ACE2 and spike protein activation [13, 14]. Thus, the cells on which ACE2 and TMPRSS2 are concurrently present are most prone to the infection of SARS-CoV [15]. Similarly, SARS-CoV-2 uses receptor ACE2 for host cell entry and cellular serine protease TMPRSS2 for S protein priming [5]. Moreover, CD147 has also been reported to functionally facilitate the cell entry of SARS-CoV-2 [16].

The angiotensin-converting enzyme 2 gene (ACE2) also known as ACEH is located on Xp22.2 and encoded by an 805-amino-acid-long protein. It belongs to the family of the angiotensin-converting enzyme of dipeptidyl carboxydipeptidases [17, 18]. It is commonly expressed in the cell membrane of the cells present in many organs such as lungs, intestine, heart, kidneys, testes, and arteries [19–21].

### SARS-CoV-2 and male reproductive system

It is now a well-known fact that males are more susceptible to SARS-CoV-2 infection than females and the fatality rate is also higher in them. The ACE2 receptors are highly expressed in testes along with other various tissues of the body making it one of the targets for SARS-CoV-2 [22]. Cells of seminiferous duct, spermatogonia, Leydig cells, and Sertoli cells are four major types of testicular cells showing high expression of ACE2 [23, 24]. ACE2 expression was found to be age-specific in males with a peak of the positive rate being 30 years of age, which indicates that SARS-CoV-2 can more likely infect young males than older ones. Moreover, the positive rate of ACE2 is higher in infertile men than normal fertile men which point to the fact that reproductive disorders are caused by ACE2-mediated pathways in SARS-CoV-2-infected men and those with reproductive disorders are more susceptible to SARS-CoV-2 infection [24].

Right from the onset of the pandemic, various studies have been published reporting the effect of the SARS-CoV-2 virus on the male reproductive system and the presence of it in male gametes. In one such report by Yang and colleagues, SARS-CoV-2 was detected in the testes from one out of 12 patients by RT-PCR, who died due to COVID-19. Also, the testes from COVID-19 patients significantly displayed seminiferous injuries, reduction in Leydig cells and mild inflammation [25]. But, due to the small sample size of the study, it is not possible to state that testicular alterations are a result of the direct or indirect effect of SARS-CoV-2 infection. A recent study suggests that since Leydig cells express ACE2, so there may be a direct correlation between the lower level of serum testosterone and inflammatory cytokines and the poor clinical outcomes among male patients infected with SARS-COV-2 [26].

The cell-type-specific expression pattern analysis of SARS-CoV-2 receptor ACE2 and entry-associated protease TMPRSS2, as well as their co-expression, confirmed the ACE2 expression in myeloid cells, spermatogonial stem cells and Leydig cells. TMPRSS2 is expressed predominantly in elongated spermatids as compared to spermatogonial stem cells. Although a relatively lesser number of spermatogonial stem cells were found to express ACE2 and TMPRSS2, cells co-expressing both genes were extremely rare (0.05%). In these cells, the alternative role of receptors and proteases was suspected to mediate viral entry [8].

The occurrence of orchitis is a general phenomenon associated with some men with SARS [27]. All SARS-infected male patients observed profuse germ cell destruction due to which there was the scarcity of spermatozoon in the seminiferous tubule and the thickness of the basement membrane increased. Besides, SARS-CoV was found in testicular epithelial cells and Leydig cells in autopsies of two patients who died of SARS [28].

Moreover, various reports indicate that SARS-CoV-2 can operate via multiple mechanisms such as inflammatory responses, oxidative stress, and apoptotic pathways, etc., to interrupt male reproductive functions [29–31]. The SARS-CoV infections result in the overproduction of reactive oxygen species that may accelerate the NF-κB–TLR (nuclear factor kappa-light chain-enhancer of activated B cells-toll-like receptor) pathways. This results in releasing of cytokines which further exaggerates the inflammatory response. Also, the orchitis induced by SARS-CoV-2 infection can lead to oxidative stress in the testis tissue [30]. This oxidative stress is known to cause intracellular oxidative damage to spermatozoa via lipid peroxidation of sperm membrane which leads to deterred semen quality and sperm functions, which in turn imparts male infertility [32].

Although till now, there are not enough shreds of evidence to prove that the male gametes may be affected by SARS-COV-2 infection, it is conceived that the spermatogenesis may be impacted due to the occurrence of fever as this process is temperature-sensitive [33]. SARS-CoV-2 has been found to induce impaired spermatogenesis and retarding sperm maturation which may be due to the immune response in testis and epididymis in male patients [34]. From February 2020 to August 2020, we have found a total of seven papers (Table 1) in which almost every study claims that no evidence of SARS-CoV-2 was found in the semen of males who recovered from COVID-19 [35–40], except for one study [41] where SARS-CoV-2 was found in the semen of six patients suffering from COVID-19 out of total 38 tested patients. This study by Li et al. raises the concern that SARS-CoV-2 is capable of surviving in the semen of infected male patients and may even persist in recovering patients. On the other hand, because of the inefficient blood-testes/deferens/epididymis barriers and the existence of systemic local inflammation in the male reproductive tract, SARS-CoV-2 may remain confined to the male reproductive tract especially in testes due to its privileged immunity [41].

**Table 1:** Various studies showing the results of semen samples and testicular tissue tested for SARS-CoV-2

Study	Date of publication	No. of samples tested		SARS-CoV-2
		Semen	Testicular biopsy	
Song et al.	April 16, 2020	13	-	Negative
Pan et al.	April 17, 2020	34	-	Negative
Paoli et al.	April 23, 2020	1	-	Negative
Li et al.	May 07, 2020	38	-	6 positive
Guo et al.	June 29,2020	23	-	Negative
Ma et al.	July 04, 2020	12	-	Negative
Holtmann et al.	August, 2020	34	-	Negative

Although SARS-CoV-2 was not detected in semen or testicular tissue in the majority of the abovementioned studies except one, still the possibility of testes being a potential target for SARS-CoV-2 cannot be ruled out because of the presence of ACE2 receptors on it. Therefore, we recommend more follow-up studies with a larger population size to investigate the reproductive function of infected as well as recovered COVID-19 male patients to benefit clinical practice and public health.

### SARS-CoV-2 and female reproductive system

Many epidemics causing viruses that are known to infect mankind in the past have been reported to affect the female reproductive system. Therefore, various researchers tried to find receptors of SARS-CoV-2 in the female reproductive organs as well as other indirect effects of SARS-CoV-2 on female reproduction.

Qiu and colleagues were the first who tried to detect SARS-CoV-2 in the vaginal fluid of postmenopausal women having COVID-19 infection. Even when the patient’s respiratory symptoms were severe, the results of vaginal swabs were found to be negative for the presence of SARS-CoV-2 [42]. Due to the sample size limitation, this study could not draw any conclusion about the presence of SARS-CoV-2 in the reproductive organs of the female. Similarly, in another report, vaginal swabs obtained from the SARS-CoV-2-infected women were found negative [43], but in one study [44], vaginal fluid of a woman tested positive on the 7th and 20th days after infection following a previous negative report at the onset of the symptoms. Based on these reports, it can be concluded that large population studies will be required to draw any conclusion about the presence of SARS-CoV-2 in the lower genital tract of females so that due precautions and preventive measures may be implemented.

Previously, ACE2 receptors were detected in the ovaries of both reproductive-aged as well as postmenopausal women [45]. Also, ACE2 is abundantly expressed in the ovaries and the oocytes. Therefore, it could be possible that SARS-CoV-2 may target the ovaries and oocytes [46]. Also, in some previous reports, ACE2 mRNA was detected in the uterus and vagina of humans [47] and rat [48]. ACE2 is generally highly expressed in epithelial cells as compared to stromal cells of the endometrium, and expression of ACE2 is elevated in the secretory phase as compared to the proliferative phase of the menstrual cycle [47]. Further, in one study, ACE2 was found highly expressed in female rats as compared to male rats and the expression gets dramatically reduced with aging in both genders [49]. Thus, based on these studies, the possibility of SARS-CoV-2 infecting the organs of the female reproductive system is much greater and thus cannot be undermined.

It can be hypothesized that SARS-CoV-2 strikes the ovarian tissue and granulosa cells and thus hampers the ovarian function and oocyte viability leading to infertility and miscarriage. It may also mutilate the endometrial cells, thus distressing the early embryo implantation [31]. Currently, due to the lack of RNAseq data available from human ovarian tissue, various researchers have employed the information available from closely related non-human primates as a substitute for human species [8, 50]. However, such studies have their limitations and cannot be considered decisive.

Furthermore, the scRNA sequencing data analysis of seven ovarian cell types including oocytes and six somatic cell types (stromal cells, granulosa cells, smooth muscle cells, natural killer cells, macrophages, and endothelial cells) from non-human primate ovary showed that the degree of ACE2 and TMPRSS2 coexpression increased with oocyte maturity in the following manner: primordial (17.0%), primary (39.0%), secondary (25.0%), and antral (62.1%). ACE2 (100% cells) is more predominantly expressed in oocytes than TMPRSS2 (37% cells) suggesting that the latter may be a potential limiting factor for infection of the female gamete. The results of this study [8] suggested that cumulus-enclosed oocytes are unlikely to be susceptible to infection by SARS-CoV-2.

The risk of SARS-CoV-2 infection increases at the time of assisted reproductive techniques to induce pregnancy. Invasive procedures such as transvaginal oocyte retrieval increase the chance of infection as the viremia is present in the SARS-CoV-2 patients [51].

Based on these observations, it is clear that we have very little information about the risk of SARS-CoV-2 infection on female reproductive organs. Also, no proper guidelines for the safety procedures during assisted reproductive technology have been put to action. It, therefore, becomes the need of the hour to put extensive research in this field to evaluate the weight of threat being posed by SARS-CoV-2 on the female reproductive system and then to design the safety measures to protect the gametes in case they are in the line of threat.

### Vertical transmission of SARS-CoV-2

It is very important to understand the risk of maternal to fetal transmission of SARS-CoV-2 to prevent the future generation from deadly SARS-CoV-2. Recently, many reports were published related to placental abnormalities in the SARS-CoV-2-infected pregnant women such as maternal vascular malperfusion of the placental bed, extensive intervillous fibrin deposition, and choriohemangioma [52–54]. Initially, it was thought that vertical transmission of SARS-CoV-2 is not possible as in some preliminary studies, the placenta was reported negative for SARS-CoV-2 [31, 53, 55]. However, many recent studies confirm the presence of SARS-CoV-2 in the placenta which supports the theory of vertical transmission of SARS-CoV-2 [56–58]. Penfield et al. have reported a study in which out of 11 placental or membrane swabs sent following delivery, 3 swabs were positive for SARS-CoV-2, all in women with moderate to severe COVID-19 illness at the time of delivery. This was the first study to demonstrate the presence of SARS-CoV-2 RNA in placental or membrane samples [59]. While there were no clinical signs of vertical transmission, their findings raise the possibility of intrapartum viral exposure.

Recently, Vivanti and colleagues reported a trans-placental transmission from a COVID-19-positive mother to her neonate. The mother was infected in the last trimester of pregnancy and the placenta was reported positive for SARS-CoV-2 genes. SARS-CoV-2 was also detected in the blood samples from the mother along with her neonate. A maximum viral load was detected in the placental tissue than in the amniotic fluid or maternal or neonatal blood. As per this report, the trans-placental transmission might result in neonatal viremia as well as placental inflammation [58].

Similarly, an Indian case study reported the strong possibility of vertical transmission of SARS-CoV-2 from a mildly symptomatic COVID-19-positive mother to her child who significantly displayed the symptoms of COVID-19 on day 2 of his life. RT-PCR test confirmed the presence of SARS-CoV-2 on the umbilical stump, placenta, and nasopharyngeal aspirate of the neonate [56]. In this case, the transmission might have occurred either via the trans-placental route or intrapartum.

In some studies, the specific antibodies such as IgG and IgM were detected in the neonates of COVID-19-positive mothers but SARS-CoV-2 was not detected in the nasopharyngeal/blood samples of the newborns [60–62]. Whereas, in many other studies, SARS-CoV-2 was found in the neonates born to COVID-19-positive mothers, but it could not be determined whether the route of transmission was placenta, the amniotic fluid, or the blood samples of both mother and fetus [63–67].

Based on all these reports, we can say that different modes of the SARS-CoV-2 transmission are being uncovered gradually and the possibility of vertical transmission cannot be denied. However, various facts limit the possibility of vertical transmission. Firstly, viremia is present in only 1% of total COVID-19 symptomatic patients which decreases the chances of maternal-fetal transmission via the placenta. The placenta has a maternal-fetal interface barrier which protects the fetus from all kind of maternal infection. Also, the immune cells of the placenta possess antiviral properties which further prevent the entry of SARS-CoV-2 [68]. Moreover, the fact that the occurrence of placental infection is very rare is further strengthened by the observation that the cell receptor for SARS-CoV-2, i.e., ACE2, is present at low levels in the human placenta during the third trimester of pregnancy [69] while there are no data on the expression of this receptor in 2nd and 3rd-trimester placentas.

Despite all these facts, we cannot deny that ACE2 receptors might present in the placenta, which increases the possibility of viral infection if the SARS-CoV-2 is present in the blood of COVID-19-positive pregnant women. Current literature is not enough to confirm vertical transmission of SARS-CoV-2; therefore, we recommend large population studies with better follow-up and data analysis for establishing further guidelines and recommendations regarding the care of pregnant women and their neonates during the current pandemic.

### Breastfeeding and COVID 19

Breastfeeding is fundamental for the infant and young child survival, nutrition and development and maternal health. The World Health Organization recommends exclusive breastfeeding for the first 6 months of life, followed by continued breastfeeding with appropriate complementary foods for up to 2 years and beyond [70]. Therefore, breastfeeding by a SARS-CoV-2-infected mother to her child becomes worrisome and raises questions of infant safety.

Pereira and colleagues observed 22 COVID-19-positive mothers and their newborns for a successive 1.8-month follow-up period. Out of all 22 women, 20 (90.9%) chose to breastfeed their babies. During the follow-up period, no neonates were infected during breastfeeding and no major complications were observed in their health. This study recommended the continuation of breastfeeding in COVID-19-positive mothers with adequate infection control measures [71].

Similarly, a WHO report also observed the breast milk samples of 46 mother-infant dyads for SARS-CoV-2 presence. All mothers were COVID-19 positive, while only 13 infants were tested COVID-19 positive. Breast milk samples from 43 mothers were negative for the COVID-19 virus while samples from 3 mothers tested positive for viral particles by RT-PCR. Out of the 3 infants whose mothers’ breast milk had viral RNA particles, but not a live virus, one infant tested positive for COVID-19 but infant feeding practices were not reported in that case. The two other infants tested negative for COVID-19; one was breastfed, and the other newborn was fed expressed breast milk after viral RNA particles were no longer detected. In the single child with COVID-19, it could not be found that through which route or source the infant got infected, i.e., through breast milk or droplet from close contact with the infected mother [72].

Furthermore, some other case reports have also validated the presence of SARS-CoV-2 in breast milk [73–75]. As per these reports, breast milk of three women was found positive for SARS-CoV-2 infection by RT-PCR out of a total of six women. Only one neonate was tested positive for COVID-19; however, it was not clear that he was infected through breast milk or some other source of infection. All infants were free of any clinical complications.

Detection of COVID-19 viral RNA in breast milk is not as much stronger evidence as the detection of the viable and infective virus would have been. Transmission of COVID-19 depends on the presence of a replicative and infectious virus that is capable of reaching the target sites in the infant and also to overcome the infant immune system. Keeping all these facts in mind, WHO recommends that COVID-19-positive mothers should be encouraged to breastfeed their children as the benefits of breastfeeding are always higher than the potential risk of SARS-CoV-2 transmission [76].

The risk of transmission based on breastfeeding should be quantified, compared, and evaluated by large sample studies keeping in mind the benefits and necessity of breastfeeding and infant nurturing interaction, only then it can be encouraged or stopped in the case of COVID-19-positive mothers.

### The psychological effect of SARS-CoV-2 on reproductive health

COVID-19 pandemic has had serious consequences on reproductive and mental health everywhere in the world. Women especially are at higher risk because of various reasons such as their traditional role as caregiver, their lack of authoritative power in their sexual and reproductive health, and increased domestic violence incidences. Reproduction and access to contraception are basic human rights. Due to the COVID-19 pandemic, the production and transportation of contraceptive commodities have been severely affected. This resulted in unintended pregnancies which also lead to stress and depression [77].

Depression and anxiety during pregnancy are associated with increased risk of preterm delivery as well as reduced cognitive and emotional development of infants [78]. Further, an Italian cross-sectional study of 100 pregnant women reported a significant psychological impact of the COVID-19 pandemic on the mental health of these women [79]. Based on the aforementioned reports, we can say that there is a strong need for intervention to assess the mental health disorders among women during the current pandemic period to avoid the overload of fear and stress.

It is expected that psychological disorders and poor male fertility are associated with each other. The decreased sperm quality and induced sexual dysfunction lead to poor fertility in males which triggers the stressors. Depressed sub-fertile men are observed to have reduced secretion of sex hormone-binding globulin and dehydroepiandrosterone sulphate is reduced in depressed sub-fertile men [80]. In addition to decreased male fertility, psychiatric distress also diminishes sexual desire and causes erectile difficulties leading to sexual inactivity. The chances of conception in the female partners of psychologically stressed men were decreased as compared to the female partners of psychologically sound men [81]. Therefore, mental health becomes equally important as physical health to improve reproductive health in the current pandemic situations.

In case the psychological effects related to COVID19 become more severe and traumatic in women, this may harm the oocyte quality and reproductive functions. In most of the IVF cycles, the lower pregnancy rates are found to be associated with the levels of adrenaline, noradrenaline, adrenocorticotrophic hormone, natural killer cells, etc., which are the common indicators of stress [82]. Several studies claim that women having post-traumatic stress disorder (PTSD) are more susceptible to various sexual dysfunctions [83–85].

## Conclusion

This review brings together the findings and observations made in the area of reproductive health during the current pandemic. Since SARS-CoV-2 is a novel virus and numerous studies concerning it are being published regularly with the advancement in research, so the data presented by upcoming new studies may contradict our statements. The presence of SARS-COV-2 in the semen of male patients rules out the fact that avoiding contact with the patient’s saliva and blood is sufficient to prevent the infection. At present, only one study supports the presence of SARS-CoV2 in the vaginal fluid. COVID-19 viral RNA has been detected in the breast milk of infected patients. Although there is not sufficient proof of risk imposed by the traces of the virus, still the risk associated with breastfeeding and infant nurturing should be elaborately studied. As far as pregnancy is concerned, severe COVID-19 cases are found to be associated with premature labor and early delivery but sufficient evidence could not be found to claim that these changes are caused by SARS-CoV-2. The reproductive system of the young population is preordained for subsequent disorders, infertility, reduced sperm count, and motility. Therefore, the research and medical practices should focus on the possible vulnerability being posed by SARS-CoV-2 on gametes and future generations.

## Data Availability

Not applicable

## Abbreviations

ACE2: Angiotensin-converting enzyme 2
ART: Assisted reproductive technology
COVID-19: Coronavirus disease 2019
NF-kB: Nuclear factor kappa-light chain-enhancer of activated B cells
PTSD: Post-traumatic stress disorder
RBD: Receptor binding domain
RNA: Ribonucleic acid
RNA seq: Ribonucleic acid sequencing
RT-PCR: Reverse transcriptase-polymerase chain reaction
scRNAseq: Single-cell RNA sequencing
SARS-CoV-2: Severe acute respiratory syndrome coronavirus 2
TLR: Toll-like receptor
TMPRSS2: Transmembrane serine protease 2

## References

[1] Wang H, Cain JG, Uraco A, Lindstrom E, Johnstone RE (2020). Getting to a new normal: mandating that patients wear masks as hospitals fully reopen during the coronavirus pandemic: comment. Anesthesiology.

[2] Coronavirus update live https://www.worldometers.info/coronavirus/. Accessed 14 September, 2020.

[3] Lukassen S, Chua RL, Trefzer T, Kahn NC, Schneider MA, Muley T, Winter H, Meister M, Veith C, Boots AW, Hennig BP, Kreuter M, Conrad C, Eils R (2020). SARS-CoV-2 receptor ACE2 and TMPRSS2 are primarily expressed in bronchial transient secretory cells. EMBO J.

[4] Yan R, Zhang Y, Li Y, Xia L, Guo Y, Zhou Q (2020). Structural basis for the recognition of SARS-CoV-2 by full-length human ACE2. Science.

[5] Hoffmann M, Kleine-Weber H, Schroeder S, Kruger N, Herrler T, Erichsen S, Schiergens TS, Herrler G, Wu NH, Nitsche A, Muller MA, Drosten C, Pohlmann S (2020). SARS-CoV-2 cell entry depends on ACE2 and TMPRSS2 and is blocked by a clinically proven protease inhibitor. Cell.

[6] Zou X, Chen K, Zou J, Han P, Hao J, Han Z (2020). Single cell RNA-seq data analysis on the receptor ACE2 expression reveals the potential risk of different human organs vulnerable to 2019-nCoV infection. Front Med.

[7] Dutta S, Sengupta P (2020) SARS-CoV-2 and male infertility: possible multifaceted pathology. Reprod Sci. 10.1007/s43032-020-00261-z 10.1007/s43032-020-00261-z PMC7351544 32651900

[8] Stanley KE, Thomas E, Leaver M, Wells D (2020). Coronavirus disease-19 and fertility: viral host entry protein expression in male and female reproductive tissues. Fertil Steril.

[9] Rabi FA, Al Zoubi MS, Kasasbeh GA, Salameh DM, Al-Nasser AD (2020). SARS-CoV-2 and coronavirus disease 2019: what we know so far. Pathogens.

[10] Cyranoski D (2020). Mystery deepens over animal source of coronavirus. Nature.

[11] Walls AC, Park YJ, Tortorici MA, Wall A, McGuire AT, Veesler D (2020). Structure, function, and antigenicity of the SARS-CoV-2 spike glycoprotein. Cell.

[12] Kuba K, Imai Y, Rao S, Gao H, Guo F, Guan B, Huan Y, Yang P, Zhang Y, Deng W, Bao L, Zhang B, Liu G, Wang Z, Chappell M, Liu Y, Zheng D, Leibbrandt A, Wada T, Slutsky AS, Liu D, Qin C, Jiang C, Penninger JM (2005). A crucial role of angiotensin converting enzyme 2 (ACE2) in SARS coronavirus–induced lung injury. Nat Med.

[13] Glowacka I, Bertram S, Muller MA, Allen P, Soilleux E, Pfefferle S, Steffen I, Tsegaye TS, He Y, Gnirss K, Niemeyer D, Schneider H, Drosten C, Pohlmann S (2011). Evidence that TMPRSS2 activates the severe acute respiratory syndrome coronavirus Spike protein for membrane fusion and reduces viral control by the humoral immune response. J Virol.

[14] Heurich A, Winkler-H H, Gierer S, Liepold T, Jahn O, Pohlmann S (2013). TMPRSS2 and ADAM17 cleave ACE2 differentially and only proteolysis by TMPRSS2 augments entry driven by the severe acute respiratory syndrome coronavirus spike protein. J Virol.

[15] Shulla A, Heald-Sargent T, Subramanya G, Zhao J, Perlman S, Gallagher T (2011). A transmembrane serine protease is linked to the severe acute respiratory syndrome coronavirus receptor and activates virus entry. J Virol.

[16] Liu C, von Brunn A, Zhu D (2020) Cyclophilin A and CD147: novel therapeutic targets for the treatment of COVID-19. Med Drug Discov 7:100056. 10.1016/j.medidd.2020.100056 PMC7364167 32835213

[17] Itoyama S, Keicho N, Hijikata M, Quy T, Phi NC, Long HT, Ha LD, Ban VV, Matsushita I, Yanai H, Kirikae F, Kirikae T, Kuratsuji T, Sasazuki T (2005). Identification of an alternative 5’-untranslated exon and new polymorphisms of angiotensin converting enzyme 2 gene: lack of association with SARS in the Vietnamese population. Am J Med Genet A.

[18] Tipnis SR, Hooper NM, Hyde R, Karran E, Christie G, Turner AJ (2000). A human homolog of angiotensin-converting enzyme. Cloning and functional expression as a captopril-insensitive carboxypeptidase. J BiolChem.

[19] Donoghue M, Hsieh F, Baronas E, Godbout K, Gosselin M, Stagliano N, Donovan M, Woolf B, Robison K, Jeyaseelan R, Breitbart RE, Acton S (2000). A novel angiotensin-converting enzyme-related carboxypeptidase (ACE2) converts angiotensin I to angiotensin 1–9. Circ Res.

[20] Gheblawi M, Wang K, Viveiros A, Nguyen Q, Zhong JC, Turner AJ, Raizada MK, Grant MB, Oudit GY (2020). Angiotensin-converting enzyme 2: SARS-CoV-2 receptor and regulator of the renin-angiotensin system: celebrating the 20th anniversary of the discovery of ACE2. Circ Res.

[21] Ohtsuki M, Morimoto S, Izawa H (2010). Angiotensin converting enzyme 2 gene expression increased compensatory for left ventricular remodeling in patients with end-stage heart failure. Int J Cardiol.

[22] Verma S, Saksena S, Sadri-Ardekani H (2020). ACE2 receptor expression in testes: implications in coronavirus disease 2019 pathogenesis dagger. Biol Reprod.

[23] Wang Z, Xu X (2020). scRNA-seq profiling of human testes reveals the presence of ACE2 receptor, a target for SARS-CoV-2 infection, in spermatogonia, Leydig and Sertoli cells. Cells.

[24] Shen Q, Xiao X, Aierken A, Liao M, Hua J (2020). The ACE2 expression in Sertoli cells and germ cells may cause male reproductive disorder after SARS-CoV-2 infection. J Cell Mol Med.

[25] Yang M, Chen S, Huang B, Zhong JM, Su H, Chen YJ, Cao Q, Ma L, He J, Li XF, Li X, Zhou JJ, Fan J, Luo DJ, Chang XN, Arkun K, Zhou M, Nie X (2020). Pathological findings in the testes of COVID-19 patients: clinical implications. Eur Urol Focus.

[26] Hussain AN, Hussain F, Hashmi SK (2020). Role of testosterone in COVID-19 patients – a double-edged sword?. Med Hypotheses.

[27] Xu J, Qi L, Chi X (2006). Orchitis: a complication of severe acute respiratory syndrome (SARS). Biol Reprod.

[28] Zhao JM, Zhou GD, Sun YL, Wang SS, Yang JF, Meng EH, Pan D, Li WS, Zhou XS, Wang YD, Lu JY, Li N, Wang DW, Zhou BC, Zhang TH (2003). Clinical pathology and pathogenesis of severe acute respiratory syndrome. Zhonghua Shi Yan He Lin Chuang Bing Du Xue Za Zhi.

[29] Sengupta P, Dutta S (2020). Does SARS-CoV-2 infection cause sperm DNA fragmentation? Possible link with oxidative stress. Eur J Contracept Reprod Health Care.

[30] Delgado-Roche L, Mesta F (2020). Oxidative stress as key player in severe acute respiratory syndrome coronavirus (SARS-CoV) infection. Arch Med Res.

[31] Li R, Yin T, Fang F, Li Q, Chen J, Wang Y, Hao Y, Wu G, Duan P, Wang Y, Cheng D, Zhou Q, Zafar MI, Xiong C, Li H, Yang J, Qiao J (2020). Potential risks of SARS-Cov-2 infection on reproductive health. Reprod Bio Med Online.

[32] Haghpanah A, Masjedi F, Alborzi S, Hosseinpour A, Dehghani A, Malekmakan L, Roozbeh J (2020). Potential mechanisms of SARS-CoV-2 action on male gonadal function and fertility: current status and future prospects. Andrologia.

[33] Huang HH, Wang PH, Yang YP, Chou SJ, Chu PW, Wu GJ, Chang CC (2020). A review of severe acute respiratory syndrome coronavirus 2 infection in the reproductive system. J Chin Med Assoc.

[34] Li H, Xiao X, Zhang J, Zafar MI, Wu C, Long Y, Lu W, Pan F, Meng T, Zhao K, Zhou L, Shen S, Liu L, Liu Q, Xiong C (2020) Impaired spermatogenesis in COVID-19 patients. EClinicalMedicine:100604 10.1016/j.eclinm.2020.100604 10.1016/j.eclinm.2020.100604 PMC7584442 33134901

[35] Song C, Wang Y, Li W, Hu B, Chen G, Xia P, Wang W, Li C, Diao F, Hu Z, Yang X, Yao B, Liu Y (2020). Absence of 2019 novel coronavirus in semen and testes of COVID-19 patients. Biol Reprod.

[36] Paoli D, Pallotti F, Colangelo S, BasilicoF,Mazzuti L, Turriziani O et al. (2020) Study of SARS-CoV-2 in semen and urine samples of a volunteer with positive naso-pharyngeal swab. J Endocrinol Investig doi:10.1007/s40618-020-01261-1. PMID: 32329026 10.1007/s40618-020-01261-1 PMC7179792 32329026

[37] Pan F, Guo J, Song Y, Li H, Patel DP, Spivak AM (2020). No evidence of SARSCoV- 2 in semen of males recovering from COVID-19. Fertil Steril.

[38] Ma L, Xie W, Li D, Shi L, Ye G, Mao Y et al (2020) Evaluation of sex-related hormones and semen characteristics in reproductive-aged male COVID-19 patients. J Med Virol. 10.1002/jmv.26259 10.1002/jmv.26259 PMC7361404 32621617

[39] Guo L, Zhao S, Li W, Wang Y, Li L, Jiang S, Ren W, Yuan Q, Zhang F, Kong F, Lei J, Yuan M (2020) Absence of SARS-CoV-2 in Semen of a COVID-19 Patient Cohort. Andrologia. 10.1111/andr.12848 10.1111/andr.12848 PMC7362062 32598557

[40] Holtmann N, Edimiris P, Andree M (2020) Assessment of SARS-CoV-2 in human semen—a cohort study. Fertil Steril 114(2) 10.1016/j.fertnstert.2020.05.028 PMC7256599 32650948

[41] Li D, Jin M, Bao P, Zhao W, Zhang S (2020). Clinical characteristics and results of semen tests among men with coronavirus disease 2019. JAMA Netw Open.

[42] Qiu L, Liu X, Xiao M, Xie J, Cao W, Liu Z, Morse A, Xie Y, Li T, Lan Zhu L (2020). SARS-CoV-2 is not detectable in the vaginal fluid of women with severe COVID-19 infection. Clin Infect Dis.

[43] Cui P, Chen Z, Wang T, Dai J, Zhang J, Ding T, Jiang J, Liu J, Zhang C, Shan W, Wang S, Rong Y, Chang J, Miao X, Ma X, Wang S (2020). Severe acute respiratory syndrome coronavirus 2 detection in the female lower genital tract. Am J Obstet Gynecol.

[44] Scorzolini L, Corpolongo A, Castilletti C, Lalle E, Mariano A, Nicastri E (2020) Comment of the potential risks of sexual and vertical transmission of Covid-19 infection. Clin Infect Dis:ciaa445. 10.1093/cid/ciaa445 10.1093/cid/ciaa445 PMC7184408 32297915

[45] Reis FM, Bouissou DR, Pereira VM, Camargos AF, Dos Reis AM, Santos RA (2011) Angiotensin-(1-7), its receptor Mas, and the angiotensin-converting enzyme type 2 are expressed in the human ovary. Fertil Steril 95:176–181. 10.1016/j.fertnstert.2010.06.060 20674894

[46] Jing Y, Run-Qian L, Hao-Ran W, Hao-Ran C, Ya-Bin L, Yang G, Fei C (2020). Potential influence of COVID-19/ACE2 on the female reproductive system. Mol Hum Reprod.

[47] Vaz-Silva J, Carneiro MM, Ferreira MC, Pinheiro SVB, Silva DA, Silva AL, Witz CA, Reis AM, Santos RA, Reis FM (2009). The vasoactive peptide angiotensin-(1—7), its receptor Mas and the angiotensin-converting enzyme type 2 are expressed in the human endometrium. Reprod Sci.

[48] Brosnihan KB, Bharadwaj MS, Yamaleyeva LM, Neves LA (2012). Decidualized pseudopregnant rat uterus shows marked reduction in Ang II and Ang-(1-7) levels. Placenta.

[49] Xudong X, Junzhu C, Xingxiang W, Furong Z, Yanrong L (2006). Age- and gender-related difference of ACE2 expression in rat lung. Life Sci.

[50] Wang S, Zheng Y, Li J, Yu Y, Zhang W, Song M (2020). Single-cell transcriptomic atlas of primate ovarian aging. Cell 180.

[51] Sadeghi MR (2020). Implications of assisted human reproduction during coronavirus disease 2019 (COVID-19) pandemic. J Reprod Infertil.

[52] Ferraiolo A, Barra F, Kratochwila C, Paudice M, Vellone VG, Godano E, Varesano S, Noberasco G, Ferrero S, Arioni C (2020). Report of positive placental swabs for SARS-CoV-2 in an asymptomatic pregnant woman with COVID-19. Medicina.

[53] Mulvey JJ, Magro CM, Ma LX, Nuovo GJ, Baergen RN (2020). Analysis of complement deposition and viral RNA in placentas of COVID-19 patients. Ann Diagn Pathol.

[54] Shanes ED, Mithal LB, Otero S, Azad HA, Miller ES, Goldstein JA (2020) Placental pathology in COVID-19. Am J ClinPathol. 10.1101/2020.05.08.20093229 10.1093/ajcp/aqaa089 PMC7279066 32441303

[55] Fan C, Lei D, Fang C, Li C, Wang M, Liu Y, Bao Y, Sun Y, Huang J, Guo Y, Yu Y, Wang S (2020) Perinatal transmission of COVID-19 associated SARS-CoV-2: should we worry? Clin Infect Dis. 10.1093/cid/ciaa226 10.1093/cid/ciaa226 PMC7184438 32182347

[56] Kulkarni R, Rajput U, Dawre R, Valvi C, Nagpal R et al (2020) Early-onset symptomatic neonatal COVID-19 infection with high probability of vertical transmission. Infection:1–5. 10.1007/s15010-020-01493-6 10.1007/s15010-020-01493-6 PMC7395939 32743723

[57] Baud D, Greub G, Favre G, Gengler C, Jaton K, Dubruc E (2020). Second-trimester miscarriage in a pregnant woman with SARS-CoV-2 Infection. JAMA.

[58] VivantiAJ V-FC, Prevot S, Zupan V, Suffee C, Cao JD, Benachi A, Luca DD (2020). Transplacental transmission of SARS-CoV-2 infection. Nat Commun.

[59] Penfield CA, Brubaker SG, Limaye MA, Lighter J, Ratner AJ, Thomas KM (2020). Detection of SARS-COV-2 in placental and fetal membrane samples. Am J Obstet Gynecol MFM.

[60] Dong L, Tian J, He S, Zhu C, Wang J, Liu C, Yang J (2020). Possible vertical transmission of SARS-CoV-2 from an infected mother to her newborn. JAMA.

[61] Zeng H, Xu C, Fan J, Tang Y, Deng Q, Zhang W, Long X (2020). Antibodies in infants born to mothers with COVID-19 pneumonia. JAMA.

[62] Zhu H, Wang L, Fang C, Peng S, Zhang L, Chang G, Xia S, Zhou W (2020). Clinical analysis of 10 neonates born to mothers with 2019-nCoV pneumonia. Transl Pediatr.

[63] Zeng L, Xia S, Yuan W, Yan K, Xiao F, Shao J, Zhou W (2020) Neonatal early-onset infection with SARS-CoV-2 in 33 neonates born to mothers with COVID-19 in Wuhan, China. JAMA Pediatr. 10.1001/jamapediatrics.2020.0878 10.1001/jamapediatrics.2020.0878 PMC7099530 32215598

[64] Yu N, Li W, Kang Q, Xiong Z, Wang S (2020). Clinical features and obstetric and neonatal outcomes of pregnant patients with COVID-19 in Wuhan, China: a retrospective, single-centre, descriptive study. Lancet Infect Dis.

[65] Alzamora MC, Paredes T, Caceres D, Webb CM, Valdez LM, La Rosa M (2020) Severe COVID-19 during pregnancy and possible vertical transmission. Am J Perinatol 37(8):861-865. 10.1055/s-0040-1710050 PMC7356080 32305046

[66] Zamaniyan M, Ebadi A, Aghajanpoor S, Rahmani Z, Haghshenas M, Azizi S (2020) Preterm delivery in pregnant woman with critical COVID -19 pneumonia and vertical transmission. Prenat Diagn. 10.1002/pd.5713 10.1002/pd.5713 PMC7264605 32304114

[67] Lorenz N, Treptow A, Schmidt S, Hofmann R, Engler MR (2020). Neonatal early-onset infection with SARS-CoV-2 in a newborn presenting with encephalitic symptoms. Pediatr Infect Dis J.

[68] Golden TN, Simmons RA (2020) Maternal and neonatal response to COVID-19. Am J Physiol Endocrinol Metab. 10.1152/ajpendo.00287 10.1152/ajpendo.00287.2020 PMC7381709 32574110

[69] Zheng QL, Duan T, Jin LP (2020). Single-cell RNA expression profiling of ACE2 and AXL in the human maternal–Fetal interface. Reprod Dev Med.

[70] World Health Organization, UNICEF (2003). Global strategy for infant and young child feeding.

[71] Pereira A, Cruz-Melguizo S, Adrien M, Fuentes L, Marin E (2020). Breastfeeding mothers with COVID-19 infection: a case series. Int Breastfeed J.

[72] Centeno-Tablante E, Medina-Rivera M, Finkelstein JL, Rayco-Solon P, Garcia-Casal MN, Ghezzi-Kopel K, Rogers L, Peña-Rosas JP, Mehta S (2020) Transmission of novel coronavirus-19 through breast milk and breastfeeding. A living systematic review of the evidence. Ann N Y Acad Sci. 10.1111/nyas.14477 10.1111/nyas.14477 PMC7970667 32860259

[73] Buonsenso D, Costa S, Sanguinetti M (2020). Neonatal late onset infection with severe acute respiratory syndrome coronavirus 2. Am J Perinatol.

[74] Wu Y, Liu C, Dong L (2020). Coronavirus disease 2019 among pregnant Chinese women: case series data on the safety of vaginal birth and breastfeeding. BJOG An Int J Obstet Gynaecol.

[75] Groß R, Conzelmann C, Müller JA (2020). Detection of SARS-CoV-2 in human breastmilk. Lancet.

[76] World Health Organization (2020). Clinical management of COVID-19: Interim guidance.

[77] Sharma A, Borah SB (2020) Covid-19 and domestic violence: an indirect path to social and economic crisis. J Fam Violence:1–7 10.1007/s10896-020-00188-8 PMC7386835 32836737

[78] Glover V (2015). Prenatal stress and its effects on the fetus and the child: possible underlying biological mechanisms. Adv Neurobiol.

[79] Saccone G, Florio A, Aiello F, Venturella R, Chiara De Angelis M, Locci M (2020). Psychological impact of COVID-19 in pregnant women. Am J Obstet Gynecol.

[80] Wdowiak A, Bień A, Iwanowicz-Palus G, Makara-Studzińska M, Bojar I (2017). Impact of emotional disorders on semen quality in men treated for infertility. Neuro Endocrinol Lett.

[81] Evans-Hoeker E, Eisenberg E, Diamond MP, Legro RS, Alvero R, Coutifaris C, Casson PR, Christman GM, Hansen KR, Zhang H, Santoro N, Steiner AZ (2018). Reproductive Medicine N. Major depression, antidepressant use, and male and female fertility. Fertil Steril.

[82] Campagne DM (2006). Should fertilization treatment start with reducing stress. Hum Reprod.

[83] Brotto L, Atallah S, Johnson-Agbakwu C, Rosenbaum T, Abdo C, Byers ES, Graham C, Nobre P, Wylie K (2016). Psychological and interpersonal dimensions of sexual function and dysfunction. J Sex Med.

[84] Mccabe MP, Sharlip ID, Lewis R, Atalla E, Balon R, Fisher AD, Laumann E, Lee SW, Segraves RT (2016). Risk factors for sexual dysfunction among women and men: a consensus statement from the Fourth International Consultation on Sexual Medicine 2015. J Sex Med.

[85] Yehuda R, Lehrner A Rosenbaum TY(2015) PTSD and sexual dysfunction in men and women. J Sex Med 12:1107–1119 10.1111/jsm.12856 25847589

